# Changes in maternal heart rate in delayed post-partum preeclampsia

**DOI:** 10.1186/s12905-023-02233-2

**Published:** 2023-03-10

**Authors:** Dorit Ravid, Michal Ovadia, Aula Asali, Shlomo Nisim, Sivan Farladansky Gershnabel, Tal Biron-Shental, Omer Weitzner

**Affiliations:** 1 Department of Obstetrics and Gynecology, Meir Medical Center, 59 Tchernichovsky St., 4428164 Kfar Saba, Israel; 2 Sackler School of Medicine, Tel Aviv University, Tel Aviv, Israel

**Keywords:** Delayed onset postpartum preeclampsia, Maternal heart rate, Maternal bradycardia, Hypertension

## Abstract

**Aim:**

Delayed-onset postpartum preeclampsia (PET) is defined as a new diagnosis of preeclampsia presenting 48 h to 6 weeks postpartum. This disorder is infrequent and associated with a higher incidence of complications as compared to antepartum PET. There seems to be a need to further characterize this disorder. The aim of the study was to investigate the difference of maternal heart rate in women with delayed onset postpartum preeclampsia as compared to healthy controls.

**Methods:**

The medical files of all women who were readmitted with delayed onset postpartum preeclampsia during 2014–2020 were reviewed. Data on maternal physiological characteristics were compared to healthy control group of women at the same post-partum day, with uncomplicated pregnancies.

**Results:**

Included 45 women with the diagnosis of delayed onset of preeclampsia at 6.3 ± 2.86 post-partum day. As compared to controls (n = 49), women with delayed post-partum were older, 34.6 ± 5.4 vs. 32.3 ± 4.7 years, *p* = 0.003. There were no differences between groups regarding maternal gravidity, parity, BMI (kg/m^2^) or Hb level at delivery day. Women with delayed post-partum preeclampsia had lower mean pulse rate as compared to controls, 58 ± 15 bpm vs. 83 ± 11.6 bpm, respectively, P < 0.0001. Only 17% of the women in the delayed onset group had pulse rate above 70 bpm as compared to 83% in the control group.

**Conclusions:**

Maternal low heart rate in cases with delayed onset of post-partum preeclampsia is an important clinical characteristic that may reflect baroreceptors response to maternal hypertension.

**Supplementary Information:**

The online version contains supplementary material available at 10.1186/s12905-023-02233-2.

## Introduction

Delayed-onset postpartum preeclampsia is defined as a new diagnosis of preeclampsia presenting 48 h to 6 weeks postpartum [1, 2]. The true incidence of de novo postpartum hypertensive disorders is not well known. Best estimates indicate that they may complicate approximately 0.15% to 2% of all pregnancies [3, 4] not including women with antepartum-onset of hypertensive disorders. Most readmissions due to postpartum hypertension occur in women without prior hypertensive disease of pregnancy [3].

The mechanism of delayed-onset preeclampsia is not known but the shared risk-factors and the similar long-term complications with antepartum preeclampsia might indicate a closely related pathophysiology. Risk-factors include obesity, older maternal age, Black race, cesarean delivery and a history of preeclampsia [1, 5]. These women tend to have greater risks for maternal morbidity, as compared to women with antepartum hypertension [5]. Based on the 2010–2014 American Nationwide Readmissions Database, Wen et al. showed that most cases of eclampsia, stroke, and overall severe morbidity associated with postpartum readmissions for hypertension occurred among women without a diagnosis of hypertensive diseases of pregnancy or of chronic hypertension, during the index delivery [3]. Moreover, long-term follow-up shows that women with delayed postpartum preeclampsia have an increased risk of progression to chronic hypertension, similar to women with antepartum preeclampsia [4].

Although delayed-onset postpartum preeclampsia shares typical clinical presentation as in antepartum preeclampsia it is commonly under-recognized and under-treated [1, 5, 6]. While looking for specific clinical characteristics to this clinical rare entity we observed that women with delayed postpartum preeclampsia had a relative sinus bradycardia as one of the clinical signs at presentation. Similar observations were reported in few case reports in both ante partum and delayed post-partum preeclampsia [6–8]. Therefore, the present study aimed to validate these observations and to compare maternal heart rate in delayed postpartum preeclampsia to normotensive women on the same postpartum day.

## Methods

We conducted a retrospective cohort study of all women readmitted to Meir Medical Center, from January 2014 through March 2020, with new delayed-onset of postpartum preeclampsia. Delayed-onset postpartum preeclampsia was defined as a new diagnosis of preeclampsia that occurred 48 h to 6 weeks postpartum. Preeclampsia was defined according to the American College of Obstetricians and Gynecologists criteria as blood pressure of 140 mm Hg systolic or 90 mm Hg diastolic or higher on two or more occasions more than 6 h apart, accompanied by proteinuria or end organ dysfunction, or blood pressure 160 mm Hg systolic or 110 mmHg diastolic or higher [9]. Excluded from the study women with prior diagnosis of preeclampsia, gestational hypertension, or chronic hypertension as well as women with prior chronic diseases.

The control group included randomly recruited healthy parturients with uncomplicated pregnancies, who came, during 2020, to the hospital for a routine screening hearing test for their newborns in the neonatal clinic, during postpartum period, on days 2–11.

.Data were collected by electronic medical record review and included: maternal age, gravity and parity, characteristics of current pregnancy (gestational age at delivery, mode of delivery, neonatal birth weight) and hemoglobin level on the day of labor. The postpartum hospitalization data included postpartum day of readmission and clinical features on presentation including pulse rate (beat per minute, bpm), blood pressure and serum laboratory values of liver function and platelet count. Pulse rate (bpm) and blood pressure of the control group were measured after at least 10 min of rest in a sitting position.

The study was approved by the Meir Medical Center Institutional Review Board on 17^th^ March 2020, number MMC-0048–20. All methods were carried out in accordance with relevant guidelines and regulations. Informed consent was not obtained from subjects due to the study nature.

### Statistical analysis

Data were analyzed using SPSS 24.0 package for windows (IBM Corp., Armonk, NY). For comparison between groups, continuous variables were analyzed with the student t test, and categorical variables were analyzed with chi-squared or Fisher’s exact test. Multivariable logistic regression was used to identify variables that were independently associated with low pulse rate in the delayed postpartum preeclampsia group. For all results, a p-value < 0.05 was considered significant.

## Results

During the study period there were 33,252 deliveries among them 45 (0.135%) women were diagnosed with delayed-onset postpartum preeclampsia.

The diagnosis of delayed onset preeclampsia was made on 6.3 ± 2.86 post-partum day. The most common symptom was headache (77.8%) followed by abdominal pain and peripheral edema (13.3% each). Shortness of breath was rare (6.7%). Abnormal laboratory tests defined as elevated liver enzymes (alanine amino transferase or aspartate amino transferase ≥ twice upper level, and /or thrombocytopenia (platelet count ≤ 100,000 /$\mu$ L), were found in 35.6% of patients. (See Table as Supplement).

Mean systolic and diastolic blood pressure levels were 162.3 ± 16.7 mmHg and 93.6 ± 10.7 mmHg, respectively. Further brain imaging tests were performed, due to maternal complaints of headache, in 35.5% of the women and seizure prophylaxis with magnesium sulphate was given to 75.5% of them.

Table 1 presents clinical and obstetrics characteristics of the delayed post-partum preeclampsia group and healthy uncomplicated controls (n = 49). As compared to controls, the delayed-onset postpartum preeclampsia group was older (34.6 ± 5.4 vs 32.3 ± 4.7, years, *p* = 0.030) and had higher cesarean delivery rate (28.8% vs 10.2%, respectively, *p* = 0.02). There were no differences between groups in maternal gravidity, parity or BMI (kg/m^2^). Pulse rates of both groups were measured at similar postpartum period: 6.3 ± 2.86 post-partum day (range 2–13) for the delayed preeclampsia group and at 5.8 ± 2.18 (range 2–11) post-partum day for the control group (*p* = 0.307) (Table 2).

**Table 1:** Maternal and obstetrics characteristics of the study groups

	Delayed preeclampsia group (n = 45)	Control group (n = 49)	*P* value
Age	34.6 ± 5.4	32.3 ± 7.4	0.030
Gravity	3.1 ± 1.3	2.8 ± 1.6	0.336
Parity	1.7 ± 1.04	1.3 ± 1.28	0.079
BMI (Kg/m^2^)	28.59	26.9	0.137
Birth weight gr	3105 ± 613	3161 ± 482	0.616
Hb	11.8 ± 1.1	11.6 ± 1.67	0.503
Cesarean delivery	28.8% (13)	10.2% (5)	0.023

Continuous variables are presented as mean ± SD and categorical variables as n (%) or median (range) as appropriate; Hemoglobin level gr% on admission to labor

**Table 2:** Maternal pulse rate of delayed onset preeclampsia group vs. normotensive postpartum control group

	Delayed preeclampsia group (n = 45)	Control group (n = 49)	*P* value
Postpartum day	6.3 ± 2.86 (2–13)	5.8 ± 2.18 (2–11)	0.307
Pulse rate/min (mean)	58 ± 15	83.3 ± 11.6	< 0.001
Pulse rate/min (median and rang)	55 (35–108)	84 (54–114)	< 0.001

Continuous variables are presented as mean ± SD and categorical variables as n (%) or median (range) as appropriate

As compared to controls the delayed-onset postpartum preeclampsia group was characterized by lower pulse rate per minute, 58 ± 15 bpm (median = 55), and 83 ± 11.6 bpm (median = 84), respectively, p < 0.001. In the delayed-onset postpartum preeclampsia group, 13(28%)  had a pulse rate of 50 bpm or less, as compared to none in the control group. 29(64%) had a pulse-rate lower than 60 as compared to 2(4%) in the control group. Only 17% of the women in the delayed-onset group had a pulse rate above 70 bpm, as compared to 83% in the control group.

Multivariable logistic regression which was used to identify clinical variables directly associated with maternal bradycardia yielded no significant correlations.

## Discussion

The present study demonstrates that women with delayed-onset postpartum preeclampsia have an extremely low heart rate as compared to healthy controls at a similar post-partum day.

The postpartum period often receives less attention than do the antenatal and intrapartum periods, and even the normal range of maternal pulse rate in the post-partum period is poorly defined [10]. Nevertheless, in a recent study by Green et al. postpartum day-specific centiles for vital signs, for the 2 weeks after birth, in “low-risk” population were established [11]. For each vital sign, a smoothed day postpartum-specific centile was calculated. Authors showed that the 50th centile heart rate was highest on the day of birth at 84 beats per minute (bpm). Median heart rate decreased progressively to the seventh day after birth, to 76 bpm, with no further significant changes in heart rate by the fourteenth postpartum day.

Observation of a significantly slow heart rate among women with delayed preeclampsia has been reported only in case reports or small series [6–8]. Most studies on delayed- onset postpartum preeclampsia concentrated mainly on clinical features. In a small series of pregnant women, it has been shown that maternal heart rate is lower in women with antenatal preeclampsia as compared to healthy controls, (71 ± 14 vs 85 ± 10 bpm), respectively [6]. Yet authors did not include patients with delayed post-partum preeclampsia.

The current study concentrated on patients with postpartum preeclampsia, which is a rare phenomenon. In concordance with previous reports [3, 4] the rate of postpartum preeclampsia was 0.135%. Women were diagnosed around the seventh day postpartum with the latest readmission occurring 21 days postpartum, and with similar rate of symptoms as headache, epigastric pain, and abnormal laboratory values [1, 5]. The main interesting observation is the relative slow pulse rate in women with delayed post-partum preeclampsia on their readmission day as compared to healthy controls. The median pulse rate was 55 bpm, which correlates with the third percentile of the cohort of Green et al. [11], as compared to 84 bmp in healthy controls, which correlates with the 65th centile on the same scale. Notably, it has been reported by another author that maternal bradycardia was a sign for increase in severity of post-partum preeclampsia with the development of HELLP syndrome [7]. These findings should be taken into consideration by the clinician in the assessment, management and chosen medications of these women.

Preeclampsia is a state of sympathetic hyperactivity [6]. As a result of this sympathetic hyperactivity usually tachycardia is observed in non-pregnant patients. Nevertheless, the current observation as described as well in case reports [7, 8] may represent dysregulation of balance in sympathetic and parasympathetic functions in women with delayed post-partum preeclampsia, leading to unexplained relative bradycardia. Additionally, Blake et al. [12] showed that changes in baroreceptor sensitivity during pregnancy were related to a withdrawal of vagal tone, rather than to an increase in sympathetic tone. These changes are reversed in the postpartum period [12]. The relative bradycardia observed by us in women with delayed-onset postpartum preeclampsia suggests that their vagal tone may have become over-active, probably baroreceptor reflex response to hypertension.

The present study is unique in several aspects. First, we validated an observation, that was reported only in case reports, of maternal slow pulse rate in postpartum preeclampsia. Second, we included only patients with new onset postpartum preeclampsia, without chronic hypertension or treated with antihypertensive medications. The current study is not without limitations. Since delayed post-partum preeclampsia is a rare disease there is a relatively small sample of patients. Moreover, additional studies on maternal cardiac evaluation were not performed.

In conclusion, women with delayed-onset postpartum preeclampsia tend to have low heart rates pulse as compared to healthy controls, on similar postpartum days. Larger studies are needed to establish this finding and for developing further recommendations for this unique group of patients.

## Supplementary Information

**Additional file 1.** Clinical presentation of delayed-onset post-partum preeclampsia group.

## Data Availability

The datasets used and/or analysed during the current study available from the corresponding author on reasonable request.
