# Supplementary material for: Changes in maternal heart rate in delayed post-partum preeclampsia
Source: BMC Womens Health. 2023 Mar 10;23:99. doi: 10.1186/s12905-023-02233-2 (PMC9999508; doi:10.1186/s12905-023-02233-2)
Supplement: Supplementary file 1 — Additional file 1. Clinical presentation of delayed-onset post-partum preeclampsia group. [file 12905_2023_2233_MOESM1_ESM.docx]

Table supplement. **Clinical presentation of delayed-onset post-partum preeclampsia group**

|  | n=45 |
| --- | --- |
| 6.3±2.86 | Days postpartum |
| **Symptoms and laboratory tests at presentation** | |
| 5(11%) | None |
| 35 (77.8%) | Headache |
| 3 (6.7%) | Shortness of breath |
| 6 (13.3%) | Epigastric pain |
| 6 (13.3%) | Peripheral edema |
| 0 | Eclampsia |
| 162.3±16.7 | Systolic BP mm Hg |
| 93.6±10.7 | Diastolic BP mm Hg |
| 17 (37.7%) | Abnormal serum laboratory values* |
| 5(11%) | Chest X-ray |
| 16(35%) | Head CT |
| 5(11%) | Brain MRI |
| 19(42%) | Intravenous antihypertensive agents |
| 23(51%) | Fundoscopy |
| 34(75%) | Magnesium-sulphate Intra-venous |
| 5.7±4.6 | Length of hospitalization (days) |

Continuous variables are presented as mean ± SD and categorical variables as n (%) or median (range) as appropriate.

*Abnormal maternal serum laboratory values at presentation included elevated liver enzymes (alanine amino transferase or aspartate amino transferase ≥ twice upper level, thrombocytopenia (platelet count ≤100,000 /$\mu$L)
